# Two new *Liolaemus* lizards from the Andean highlands of Southern Chile (Squamata, Iguania, Liolaemidae)

**DOI:** 10.3897/zookeys.632.9528

**Published:** 2016-11-16

**Authors:** Jaime Troncoso-Palacios, Hugo A. Diaz, German I. Puas, Edvin Riveros-Riffo, Alvaro A. Elorza

**Affiliations:** 1 Programa de Fisiologia y Biofisica, Instituto de Ciencias Biomedicas (ICBM), Facultad de Medicina, Universidad de Chile, Independencia 1027, Santiago, Chile; 2 Departamento de Ciencias Ecologicas, Facultad de Ciencias, Universidad de Chile, Las Palmeras 3425, Santiago, Chile; 3 Departamento de Ciencias Biologicas Animales, Facultad de Ciencias Veterinarias y Pecuarias, Universidad de Chile, Santa Rosa 11735, Santiago, Chile; 4 Centro de Investigaciones Biomedicas, Facultad de Ciencias Biologicas y Facultad de Medicina, Universidad Andres Bello, Republica 239, Santiago, Chile; 5 Instituto Milenio de Inmunologia e Inmunoterapia, Portugal 49, Santiago, Chile; 6 Red de Observadores de Aves y Vida Silvestre de Chile, Julio Prado 1144 Dpto. 31, Providencia, Chile

**Keywords:** Cytochrome b, Liolaemus elongatus, Liolaemus villaricensis, mtDNA, new species, precloacal pores

## Abstract

*Liolaemus* is a diverse genus of lizards, subdivided into two subgenera: *Liolaemus* (*sensu stricto*) and *Eulaemus*, distributed mainly in Chile and Argentina. The *Liolaemus elongatus-kriegi* complex is the most diverse group within *Liolaemus* (*sensu stricto*), especially the species closely related to *Liolaemus elongatus*, which form a clade currently comprising nine species. Several Chilean species of this group have been recently described, mainly from volcanoes and poorly explored mountains. Here molecular and morphological evidence are provided for a new species of the *Liolaemus elongatus* clade, which is characterized by its small size and lack of dorsal pattern, unusual features for the species of this group of lizards. Additionally, the lack of precloacal pores in males of *Liolaemus* (*sensu stricto*) is a trait found in few species, which do not constitute a monophyletic group. A second new southern Chilean species is also described, without precloacal pores and supported by molecular phylogenetics to be related to *Liolaemus villaricensis*. Both new species were found in the same locality, near a lake located in a pre-Andean zone with *Araucaria* and *Nothofagus* forest. The two species are dedicated to prominent Lonkos (tribal chiefs) of the Mapuche and Pehuenche people: Janequeo and Leftraru. Additionally, the phylogenetic results suggest that *Liolaemus lonquimayensis* is a synonym of *Liolaemus elongatus*.

## Introduction

*Liolaemus* is one of the most diverse genera of lizards, including 252 species (Uetz and Hošek 2015) that are grouped into two subgenera: *Liolaemus* (*sensu stricto*) and *Eulaemus* (*e.g.* Laurent 1985, Schulte et al. 2000), distributed mainly in Chile and Argentina (Abdala and Quinteros 2014). Recently, the exploration of volcanoes and rarely visited highlands in central and southern Chile has led to the discovery and description of several new species of lizards (Escobar-Huerta et al. 2015, Esquerré et al. 2013, 2014, Troncoso-Palacios et al. 2015, 2016), most of them belonging to the *Liolaemus elongatus-kriegi* complex of the *Liolaemus* subgenus. This complex consists of four clades: the *punmahuida*, *petrophilus*, *kriegi* and *elongatus* groups (Avila et al. 2004, 2012, Morando et al. 2003). The *Liolaemus elongatus* clade was characterized by Avila et al. (2015) as a group of lizards of medium to large size, having long-tails, with reduced sexual dichromatism, viviparous, insectivorous, and almost exclusively saxicolous. Currently, this clade comprises nine species distributed in central and southern Chile and Argentina: *Liolaemus antumalguen* Avila, Morando, Perez & Sites, 2010; *Liolaemus burmeisteri* Avila, Pérez, Medina, Sites & Morando, 2012; *Liolaemus carlosgarini* Esquerré, Núñez & Scolaro, 2013; *Liolaemus choique*; *Liolaemus elongatus* Koslowsky, 1896; *Liolaemus shitan*; *Liolaemus smaug* Abdala, Quinteros, Scrocchi & Stazzonelli, 2010; *Liolaemus crandalli* Avila, Medina, Fulvio-Pérez, Sites & Morando, 2015; and *Liolaemus lonquimayensis* Escobar-Huerta, Santibáñez-Toro & Ortiz, 2015. Although several new species have been described in recent years, it has been suggested that the diversity within the *Liolaemus elongatus-kriegi* complex is underestimated and the number of species could be doubled (Morando et al. 2003).

The males of most *Liolaemus* species have precloacal pores (Esquerré et al. 2013) and these are extensively used as taxonomic characters and for sex determination (Lobo 2005; Valdecantos et al. 2014). In fact, Esquerré et al. (2013) listed only five species of *Liolaemus* (*sensu stricto*) which lack precloacal pores, to which can now be added two newly described species: *Liolaemus chavin* Aguilar, Wood, Cusi, Guzmán, Huari, Lundberg, Mortensen, Ramírez, Robles, Suárez, Ticona, Vargas, Venegas & Sites, 2013, and *Liolaemus tregenzai* Pincheira-Donoso & Scolaro, 2007.

In a field campaign to southern Chile in January 2014, two sympatric species of *Liolaemus* were found which cannot be assigned to any known species. Here molecular and morphological evidence for a new species of the *Liolaemus elongatus* clade are provided. Molecular and morphological evidence are also given for another new species of *Liolaemus* (*sensu stricto*), which is the first species closely related to *Liolaemus villaricensis* Müller & Hellmich, 1932, based on molecular phylogeny.

## Materials and methods

*Morphological data and analyses*. Morphological characters were examined according to Etheridge (1995), Lobo (2005) and Avila et al. (2010, 2012). Body measurements were made with a digital vernier caliper (0.02 mm precision). Measurements are provided as mean ± standard deviation (x ± SD). Scales were observed with different magnifying lenses. The scale characterization and measurements were recorded on the right side of the specimen, unless otherwise indicated. Dorsal scales were counted between the occiput and the level of the anterior border of the hind limbs. Ventral scales were counted from mental scale to the anterior margin of the cloacal opening. Stomach and intestinal contents were analyzed under a binocular stereoscope for one specimen of each new species. Specimens were collected in four field campaigns: January 2014, February 2015, January 2016 and September 2016. Both species are characterized by their low abundance and, to our knowledge, by highly restricted distributions. Despite four field campaigns, we only were able to collect seven specimens for each new species. All specimens were sexed through internal examination of testes or oocytes/embryos. We examined specimens of all Chilean species currently considered within the *Liolaemus elongatus* clade, including nine adult specimens of *Liolaemus carlosgarini*, six adult specimens of Liolaemus cf. elongatus and eleven adult specimens of *Liolaemus scorialis*; plus six adult specimens of *Liolaemus villaricensis* Müller & Hellmich, 1932. Additional data for eight adult specimens of *Liolaemus carlosgarini* were taken from Esquerré et al. (2013) and additional data for two adult specimens of *Liolaemus villaricensis* were taken from literature (Hellmich 1934). The specimens examined are listed in Suppl. material 1: Appendix I. Acronyms used in this work are: MZUC (Colección del Museo de Zoología de la Universidad de Concepción), MRC (Museo de Historia Natural de Concepción) and SSUC (Colección Patricio Sánchez Reyes, Pontificia Universidad Católica de Chile). Data for all species endemic to Argentina were taken from literature as follow. *Liolaemus choique*; *Liolaemus shitan*; and *Liolaemus smaug* was taken from Abdala et al. (2010). Data for *Liolaemus antumalguen* were taken from Avila et al. (2010), data for *Liolaemus burmeisteri* was taken from Avila et al. (2012) and data for *Liolaemus crandalli* were taken from Avila et al. (2015). Data for *Liolaemus coeruleus* Cei & Ortiz, 1983; and *Liolaemus neuquensis* Müller & Hellmich, 1939; were taken from Scolaro et al. (2007). Data for *Liolaemus punmahuida* Avila, Pérez & Morando, 2003; were taken from Avila et al. (2003). Data for *Liolaemus tregenzai* were taken from Pincheira-Donoso and Scolaro (2007). For the diagnosis, we performed a statistical analysis with data taken from all adult specimens directly examined (Suppl. material 1: Appendix I) plus data taken from published data set of *Liolaemus antumalguen*, *Liolaemus carlosgarini* and *Liolaemus lonquimayensis*. For the statistical analysis, we applied a Kolmogorov-Smirnov test to verify data normality, a subsequent t-test or Mann-Whitney U test was used if data passed or failed the normality test, respectively, to compare each variable. The statistical results are provided only when the differences were significant. Additionally, we performed a principal component analysis (PCA) to visualize and discriminate species in the morphological space, using the following variables: head length, head width, head height, snout-vent length (SVL), axilla-groin distance (AGD), arm length, foot length, midbody scales (SAMB), dorsal scales, ventral scales, supralabial scales, infralabial scales and fourth toe lamellae. This was performed with FactoMineR and R6 packages in RStudio and missing data were previously imputed with missMDA (RStudio Team 2015). Eigenvalues and the correlation of each variable with each of the first three PCs are provided in Appendices III and IV. For species that we did not examine and for which no published data sets exist, we performed a diagnosis based in the scale count ranges and SVL range, following the diagnoses previously published for the descriptions of *Liolaemus* included in this work (Abdala et al. 2010, Avila et al. 2010, 2012, 2015, Escobar-Huerta et al. 2015, Esquerré et al. 2013). Color pattern features were used as qualitative features of diagnosis between the two new species and all related species.

*Genomic DNA purification, PCR amplification, and Sequencing*. Samples from liver and thigh muscle were obtained from ethanol-fixed lizards and subjected to a rehydration process according to Coura (2005). Samples were washed twice in distilled water for 5 min at 55 °C to remove the fixative and then rehydrated with 1x Tris/EDTA for 5 min at 55 °C and then with 1M Tris pH 7.5, at 55 °C overnight, followed immediately by digestion with proteinase K (20 mg/ml) at 55 °C overnight. Genomic DNA isolation (mitochondrial and nuclear) was done with the Wizard® Genomic DNA Purification kit (Cat # A1120, Promega, USA) following manufacturer´s instructions. The mitochondrial gene *Cyt-b* was amplified from total DNA through two phase conventional PCR with the primers GLUDGL (5´-TGA CTT GAA RAA CCA YCG TTG-3´) and CB3 (5´-GGC AAA TAG GAA RTA TCA TTC-3´), reported in Torres- Pérez et al. (2009), to generate a 665 bp amplicon. PCR reactions were performed with the SapphireAmp® Fast PCR Master Mix (Cat # RR350A, Takara Clontech, USA) using 100 ng of total genomic DNA as a template and following the instruction manual. Two-phase PCR cycling was as follow: Phase 1, initial 98 °C denaturation for 3 min, then 5 cycles of 98 °C denaturation for 30 s, 47 °C annealing for 45 s and 72 °C extension for 45 s. The Phase 2, next 40 cycles of 98 °C denaturation for 30 s, 58 °C annealing for 45 s and 72 °C extension for 45 s. A final 72 °C extension step for 5 min was added to finish the PCR. The 665 bp PCR amplicon was checked by DNA electrophoresis on a 1% agarose gel in 1× Tris-Acetate-EDTA (TAE) buffer. The amplicons were purified with the E.Z.N.A.® Cycle-Pure Kit (Cat # D6492-02, Omega Biotek, USA) and sent for capillary sequencing to Macrogen, Korea.

*Phylogenetic reconstruction*. The GenBank accession numbers of the *Cyt-b* mitochondrial loci sequences generated in this study and the sequences obtained from GenBank are indicated in Suppl. material 1: Appendix II. Additionally, Gustavo Escobar-Huerta sent us the *Cyt-b* sequences of the type series of *Liolaemus lonquimayensis* (MZUC 40365–68). Cesar Aguilar and Jack Walter Sites Jr. sent us the *Cyt-b* sequence of one of the two specimens of *Liolaemus* sp.2 included in the phylogeny (SSUC Re 716). One hundred sixteen nucleotide sequences used in the analysis were aligned using MUSCLE (Edgar 2004). JModelTest v2.1.7 (Darriba et al. 2012, Guidon and Gascuel 2003) was used to select an appropriate substitution model (HKY + G + I), based on both the BIC and AIC indices. Bayesian inference (BI) analyses were performed with MrBayes v3.1.5 (Ronquist and Huelsenbeck 2003). Two independent analyses, each consisting of two groups of four chains that ran independently, were run for 10.0 × 10^6^ generations and at sample frequency of 1000 using default priors. *Phymaturus vociferator* Pincheira-Donoso, 2004, was selected as the outgroup. Twenty-five percent of samples were discarded as burn-in when calculating the convergence diagnostic, assessed by examining values of average standard deviation of the Potential Scale Reduction Factor (PSRF = 1.000 for all parameters) (Gelmar and Rubin 1992) and the minimum and average Estimated Sample Size (ESS > 4000 for all parameters). The nodes were considered as strongly supported when pp ≥ 0.95 (Huelsenbeck and Ronquist 2001). Additionally, a maximum likelihood phylogenetic analysis (ML) was performed with 1000 bootstrap replicates and calculated the average uncorrected pairwise difference (p-distance) using MEGA v6.06 (Tamura et al. 2013). Nodes with a bootstrap value ≥ 95% were considered as strongly supported (Felsenstein and Kishino 1993).

## Results

**Figure 1.**
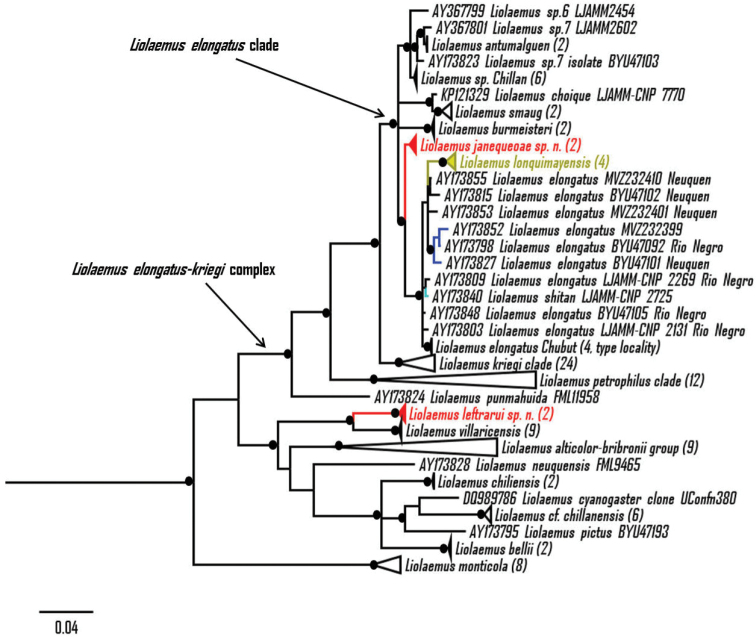
Bayesian inference of phylogeny (BI) tree based on *Cyt-b*, showing phylogenetic relationships of *Liolaemus janequeoae* sp. n. and *Liolaemus leftrarui* sp. n. (in red) (HKY+G+I). *Liolaemus shitan* is in light green, *Liolaemus lonquimayensis* is in yellow and *Liolaemus elongatus* samples used by Escobar-Huerta et al. (2015) are in blue. Posterior probability ≥ 0.95 are indicated with a black dot. Numbers in parentheses indicate the amount of collapsed sequences. Scale shows the number of changes per site.

**Figure 2.**
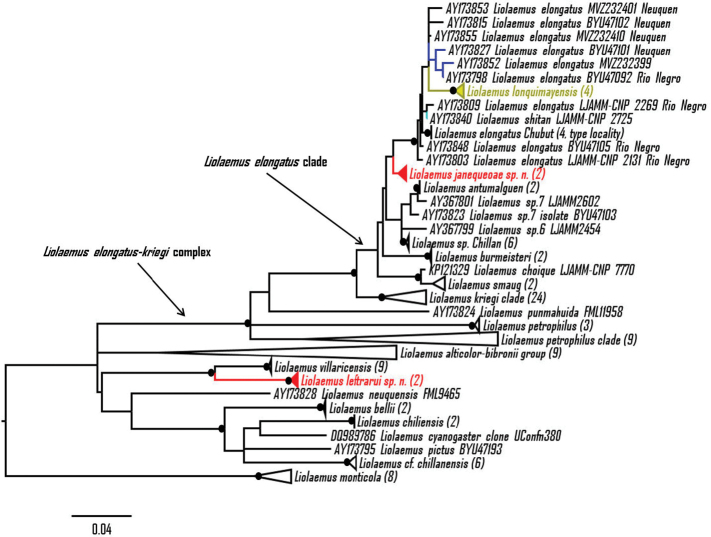
Maximum likelihood phylogeny (ML) tree based on *Cyt-b*, showing phylogenetic relationships of *Liolaemus janequeoae* sp. n. and *Liolaemus leftrarui* sp. n. (in red) (HKY+G+I). *Liolaemus shitan* is in light green, *Liolaemus lonquimayensis* is in yellow and *Liolaemus elongatus* samples used by Escobar-Huerta et al. (2015) are in blue. Bootstrap value ≥ 95% are indicated with a black dot. Numbers in parentheses indicate the amount of collapsed sequences. Scale shows the number of changes per site.

In our BI phylogeny (Fig. 1), the first species described in this work is found to be a member of the *Liolaemus elongatus* clade, which is strongly supported (pp = 0.99) and includes *Liolaemus antumalguen*, *Liolaemus burmeisteri*, *Liolaemus choique*, *Liolaemus elongatus*, *Liolaemus lonquimayensis*, *Liolaemus shitan*, *Liolaemus smaug*, the species described here, an unidentified *Liolaemus* from Chillán and two candidate species (*Liolaemus* sp. 6 and *Liolaemus* sp. 7) previously suggested by Morando et al. (2003). In the ML phylogeny (Fig. 2) the *Liolaemus elongatus* clade was recovered with moderate support (bootstrap = 88%) but with the same composition. In both analyses the first species described here is found as sister taxon of the clade *Liolaemus elongatus* + *Liolaemus lonquimayensis* + *Liolaemus shitan* (pp = 0.99 and bootstrap = 74%, respectively). The topology found in the ML phylogeny (Fig. 2) is very similar to the topology found in the BI phylogeny, but curiously *Liolaemus petrophilus* was found outside of the remainder of the *Liolaemus petrophilus* clade, which has low support (bootstrap = 48%). In both, ML and BI phylogenies, *Liolaemus shitan* and *Liolaemus lonquimayensis* appear to be conspecific with *Liolaemus elongatus*; and also *Liolaemus* sp. 7 and *Liolaemus antumalguen* appear to be conspecifics. The addition of other species of the *elongatus* clade to the phylogeny might resolve these issues (see Discussion). Average uncorrected pairwise distance between the first new species and the clade *Liolaemus elongatus* + *Liolaemus lonquimayensis* + *Liolaemus shitan* is 3.4%, consistent with a 3% divergence previously proposed for identification of candidate species in *Liolaemus* (Breitman et al. 2012).

The second species described here is found to be the sister species of *Liolaemus villaricensis* in both analysis (BI pp = 1.00, ML bootstrap = 99%, Figs 1 and 2), being the first species identified as closely related to this taxon based on molecular phylogeny. Average uncorrected pairwise distance between the species is 7.3%, more than double that value proposed for identification of candidate species (Breitman et al. 2012).

**Figure 3.**
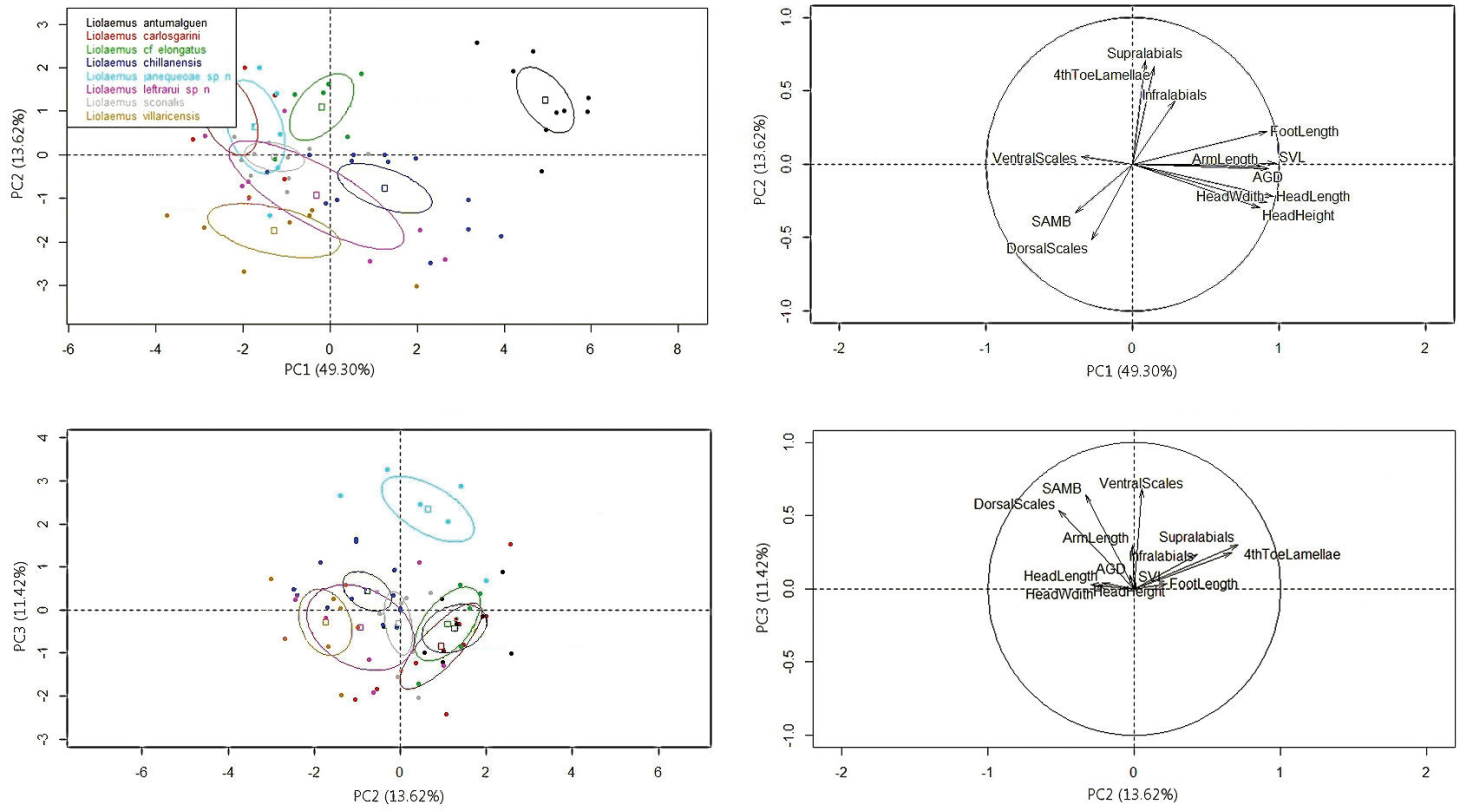
Principal Component Analysis (PCA) results. On the left, ellipses representing the 95% confidence interval around the centroid for each species. Axis correspond to the percentage of the total variance that each PC explains. On the right, contribution of each variable to the construction of the axes.

In regards to the PCA analysis, only the first three principal components (PCs) account each more than 10% of the variation (Suppl. material 1: Appendix III). PC1 is mainly positively correlated with variation in morphological measures (SVL, head length, head width, axilla-groin distance and foot length, Suppl. material 1: Appendix IV, Fig. 3). PC2 is mainly positively correlated with the number of supralabials, fourth toe lamellae, infralabials and negatively correlated to the dorsal scales (Suppl. material 1: Appendix IV, Fig. 3). PC3 is positively correlated mainly with the ventral, midbody and dorsal scale counts. The first species described here does not overlap in morphological space with *Liolaemus elongatus* (Fig. 3), found as its most closely related species in both BI and ML phylogenies (Figs 1 and 2). The second species described here marginally overlaps with *Liolaemus villaricensis* in morphological space when ellipses (95% confidence interval around the centroid for each species) are generated with the first two PCs (Fig. 3). However, the second new species overlaps almost completely in morphological space with *Liolaemus villaricensis*, *Liolaemus* cf. *chillanensis* and *Liolaemus scorialis* when ellipses are generated with the second and third PCs (Fig. 3). Nevertheless, the second species described here is not closely related to *Liolaemus* cf. *chillanensis* (Figs 1 and 2) or *Liolaemus scorialis*, a member of the *Liolaemus elongatus-kriegi* clade (D. Esquerré, pers. comm.). Additionally, the morphological and coloration differences between the second species described here and *Liolaemus villaricensis* (its sister species), and the uncorrected pairwise difference between them justify the description of this as a new species.

### Liolaemus janequeoae sp. n.

Taxon classificationAnimaliaSquamataLiolaemidae

http://zoobank.org/35D080AB-AD1F-4ED5-99E5-CEF925C539FD

Proposed standard English name: Janequeo’s Lizard

Proposed standard Spanish name: Lagarto de Janequeo

Figure 4

#### Holotype.

**Figure 4.**
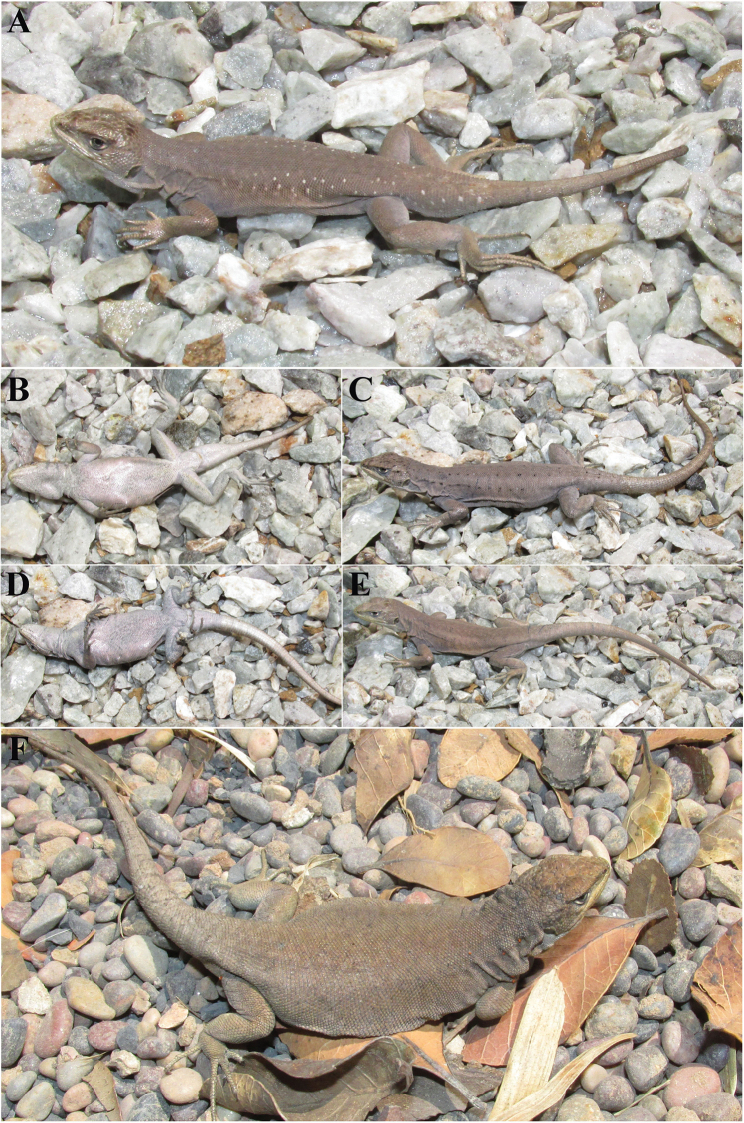
*Liolaemus janequeoae* sp. n. **A** and **B** Holotype, male **C** and **D** Paratype, female with dorsal black dots **E** and **F** Paratypes, typical females.

SSUC Re 712 (Fig. 4). Male collected at Laguna Verde (38°12'S - 71°44'W, 1397 masl), approximately 13.5 km NW of the summit of the Tolhuaca Volcano, Araucanía Region, Chile. Collected by J. Troncoso-Palacios and Edvin Riveros-Riffo. January 15, 2016.

#### Paratypes.

SSUC Re 713–14. Two females (Fig. 4). Same data as the holotype. SSUC Re 715. Female. Collected at the locality of the holotype by Edvin Riveros-Riffo. February 18, 2015. SSUC Re 649–51, three females. Collected at the locality of the holotype by J. Troncoso-Palacios, F. Urra and H. Díaz. January 5, 2014 (Fig. 4).

#### Diagnosis.

*Liolaemus janequeoae* belongs to the *Liolaemus elongatus* clade. This species is characterized by 1) small size (maximum snout vent length = 69.6 mm), 2) lack of dorsal pattern, 3) high number of midbody scales (82–98), 4) precloacal pores present in males, and 5) absence of dark rings on the tail. We provide a differential diagnosis with regards to all species currently considered to be members of this clade, plus *Liolaemus scorialis* Troncoso-Palacios Díaz, Esquerré & Urra, 2015, the assignment of which is under study, but probably is related to the *Liolaemus elongatus* clade (Troncoso-Palacios et al. 2015). Table 1 summarizes some of the diagnostic traits. Based on seven specimens.

**Table 1.** Scale count and morphological characteristics for *Liolaemus janequeoae* sp. n. and the geographically proximate species of the *Liolaemus elongatus* clade plus *Liolaemus leftrarui* sp. n. Juvenile specimens examined are excluded. Source of data for non-examined species are: *Liolaemus antumalguen* (Avila et al. 2010), *Liolaemus burmeisteri* (Avila et al. 2012) and *Liolaemus lonquimayensis* (Escobar-Huerta et al. 2015). M = males; F = females.

	*Liolaemus antumalguen*	*Liolaemus burmeisteri*	*Liolaemus carlosgarini* (M = 4, F = 5)	Liolaemus cf. elongatus (M = 3, F = 3)	*Liolaemus janequeoae* sp. n. (M = 1, F = 6)	*Liolaemus leftrarui* sp. n. (M = 3, F = 4)	*Liolaemus lonquimayensis*	*Liolaemus scorialis* (M = 8, F = 3)
Max SVL (mm)	107.8	85.2	68.8	73.3	69.6	81.8	69.7	69.9
Midbody scales range	72–82	70–81	80–95	76–88	82–98	80–88	?	76–90
Ventral scales	105–118	99–110	112–124	119–129	124–132	110–123	?	115–131
Dorsal scales	70–78	76–85	68–82	67–73	77–89	77–87	?	72–81
Dorsal pattern	Variable, from patternless to two dorsolateral series of black ocelli sometimes fused longitudinally	Light brown speckled with white spots, flanked by band of dark brown between axilla and groin, with few white spots	Marked or inconspicuous dark occipital band and dark lateral bands	Marked dark occipital band and dark lateral bands	Absent/White dots/Black dots	Dispersed bluish dots with light green in the flanks	Marked dark occipital band and dark lateral bands	Marked dark occipital band and dark lateral bands
Ventral melanism	Present	Absent	Absent	Absent to partial	Absent	Absent	Absent	Absent
Head color	Variable, from completely black to light-tan or ochre	Ochre	Light brown	Dark brown	Light brown	Brown	Brown	Brown/Light brown
Body color	Light gray to ochre	Light brown/khaki	Yellowish brown or light brown	Almost black	Light brown	Brown	Brown	Brown/Gray
Tail rings	Absent	Weak	Marked/Weak	Marked/Weak/ Absent	Absent	Absent/ Weak	Marked	Marked
Precloacal pores in males	3–4	0–5	0–3	1–5	3	0	0	3–4

*Liolaemus janequeoae* is closely related to *Liolaemus elongatus*. However, *Liolaemus janequeoae* is smaller (maximum SVL = 69.6 mm, n = 7 adults, vs. max. SVL = 94.7 mm) and has more midbody scales (82–98 vs. 68–87) than *Liolaemus elongatus* from Argentina (Table 1). Dorsal color pattern in *Liolaemus elongatus* is highly variable from vertebral and lateral dark bands to complete melanism, whereas *Liolaemus janequeoae* never has black spots (only small black dots in one female). Interestingly, SVL of *Liolaemus* cf. *elongatus* from Llaima, Chile (SVL = 68.4 ± 2.9 mm), is not significantly different compared with the SVL of *Liolaemus janequeoae* (SVL = 65.3 ± 3.4 mm); but head height is lower in *Liolaemus janequeoae* than in *Liolaemus* cf. *elongatus* (6.8 ± 0.5 mm vs 8.3 ± 0.7 mm) (t = -4.6, DF = 11, P < 0.01); the head is wider in *Liolaemus* cf. *elongatus* than in *Liolaemus janequeoae* (12.7 ± 0.9 mm vs 11.0 ± 0.4 mm) (Mann–Whitney U = 0.001, P < 0.01); *Liolaemus janequeoae* has more midbody scales than *Liolaemus* cf. *elongatus* (82–98 vs. 76–88) (t = 3.0, DF = 11, P < 0.05), more dorsal scales (77–89 vs. 67–73) (t = 7.7, DF = 11, P < 0.01) and more ventral scales (124–132 vs. 119–129) (t = 2.5, DF = 11, P < 0.05). Additionally, PCA results show that *Liolaemus janequeoae* and *Liolaemus* cf. *elongatus* from Llaima occupy a different region of morphological space, without overlap (Fig. 3).

*Liolaemus janequeoae* is smaller (SVL = 65.3 ± 3.4 mm) than *Liolaemus antumalguen* (SVL = 95.0 ± 6.2 mm) (t = -11.3, DF = 14, P < 0.01); has a shorter axilla-groin distance (27.8 ± 2.9 mm vs 43.0 ± 4.4 mm) (Mann–Whitney U, P < 0.01); a shorter arm length (24.7 ± 2.3 mm vs 28.4 ± 0.7 mm) (t = -4.5, DF = 14, P < 0.01); a lower head height (6.8 ± 0.5 mm vs 10.0 ± 0.6 mm) (t = -11.2, DF = 14, P < 0.01); a narrower head (11.0 ± 0.4 mm vs 16.6 ± 0.8 mm) (t = -17.2, DF = 14, P < 0.01); and has shorter foot length (19.4 ± 1.4 mm vs 28.5 ± 1.2 mm) (Mann–Whitney U, P < 0.01); whereas *Liolaemus janequeoae* has more midbody scales than *Liolaemus antumalguen* (t = 6.2, DF = 14, P < 0.01, Table 1), more dorsal scales (t = 7.6, DF = 14, P < 0.01, Table 1) and more ventral scales (t = 8.2, DF = 14, P < 0.01, Table 1). Moreover, *Liolaemus antumalguen* has a very variable dorsal pattern of black spots to almost complete melanism, whereas *Liolaemus janequeoae* never has black spots (only small black dots in one female). Additionally, PCA results show that both species occupy a different region of morphological space, without overlap (Fig. 3).

*Liolaemus carlosgarini*, *Liolaemus scorialis* and *Liolaemus lonquimayensis* have dark lateral and vertebral bands, features that distinguishes these from *Liolaemus janequeoae*. Additionally, *Liolaemus janequeoae* is larger than *Liolaemus carlosgarini* (SVL = 65.3 ± 3.4 mm vs SVL = 60.2 ± 5.1 mm) (t = 2.4, DF = 22, P < 0.05); *Liolaemus janequeoae* has a larger axilla-groin length than *Liolaemus carlosgarini* (27.8 ± 2.9 mm vs 24.8 ± 2.9 mm) (t = 2.3, DF = 22, P < 0.05); *Liolaemus janequeoae* has longer arms than *Liolaemus carlosgarini* (24.7 ± 2.3 mm vs 21.8 ± 1.8 mm) (t = 3.4, DF = 22, P < 0.01); *Liolaemus janequeoae* has more dorsal scales than *Liolaemus carlosgarini* (t = 4.5, DF = 14, P < 0.01, Table 1) and more ventral scales (t = 6.8, DF = 14, P < 0.01, Table 1); whereas *Liolaemus lonquimayensis* has larger axilla-groin length (34.9 ± 1.7 mm) than *Liolaemus janequeoae* (Mann–Whitney U, P < 0.05); *Liolaemus lonquimayensis* has a greater head height than *Liolaemus janequeoae* (8.3 ± 0.1 mm vs 6.8 ± 0.5 mm) (t = -4.8, DF = 8, P < 0.01); whereas *Liolaemus scorialis* has the head wider than *Liolaemus janequeoae* (11.9 ± 0.6 mm vs 11.0 ± 0.4 mm) (t = -3.1, DF = 16, P < 0.01); *Liolaemus janequeoae* has more midbody scales than *Liolaemus scorialis* (t = 3.6, DF = 16, P < 0.01, Table 1) and more dorsal scales (t = 4.8, DF = 17, P < 0.01, Table 1). Additionally, PCA results show that *Liolaemus janequeoae* does not overlap in the morphological space with *Liolaemus carlosgarini* and *Liolaemus scorialis* when ellipses are generated with the second and third PCs (Fig. 3).

*Liolaemus janequeoae* is smaller (max. SVL = 69.6 mm) than *Liolaemus shitan* (max. SVL = 98.3 mm) and has more midbody scales (82–98 vs. 72–85). Dorsal color pattern in *Liolaemus shitan* is black, whereas only one female of our sample of *Liolaemus janequeoae* has small dorsal black dots.

*Liolaemus janequeoae* is smaller (max. SVL = 69.6 mm) than *Liolaemus choique* (max. SVL = 90.7 mm). Moreover, *Liolaemus choique* has a very variable dorsal pattern of black spots to almost complete melanism, whereas *Liolaemus janequeoae* never has black spots (only small black dots in one female).

*Liolaemus janequeoae* is smaller than *Liolaemus crandalli* (max. SVL = 69.6 mm vs max. SVL = 93.4 mm). Moreover, *Liolaemus crandalli* has dark lateral and vertebral bands with ringed tail, whereas all of these features are completely absent in *Liolaemus janequeoae*. According to Avila et al. (2015), *Liolaemus crandalli* is the sister taxon of the pair *Liolaemus smaug* + *Liolaemus choique*, whereas in our phylogeny *Liolaemus janequeoae* is not closely related to *Liolaemus smaug* or *Liolaemus choique*.

*Liolaemus janequeoae* is smaller than *Liolaemus burmeisteri* (max. SVL = 69.6 mm vs max. SVL = 85.2 mm) and has more midbody (82–98 vs. 70–81) and ventral scales (124–132 vs. 99–110). Moreover, *Liolaemus burmeisteri* has dark lateral bands.

*Liolaemus janequeoae* has more midbody scales than *Liolaemus smaug* (82–98 vs 73–80). Moreover, *Liolaemus smaug* has dark lateral and vertebral band. In our phylogeny *Liolaemus janequeoae* and *Liolaemus smaug* are not sister taxa.

#### Description of holotype.

Adult male. SVL: 59.1 mm. Tail length: 42.0 mm (autotomized). Axilla-groin length: 21.8 mm. Head length: 13.1 mm. Head width (distance between the two ear openings): 10.5 mm. Head height (at the level of ear openings): 6.1 mm. Forelimb length: 21.1 mm. Hindlimb length: 36.0 mm. Foot length: 18.6 mm. Hand length: 9.8 mm. Rostral scale wider (2.36 mm) than high (0.8 mm). Subocular length: 4.2 mm. Fifth supralabial length: 1.6 mm. Neck width: 9.4 mm. Interorbital distance: 4.5 mm. Internasal distance: 1.5 mm. Body width: 13.7 mm. Meatus width: 1.4 mm. Meatus height: 2.1 mm.

Two postrostrals. Four internasals. Hexagonal interparietal scale, with a central, small, and whitish ‘‘parietal eye’’ in the center. Interparietal smaller than the parietals, surrounded by other nine scales; ten scales between interparietal scale and rostral; seventeen scales between occiput and rostral (Hellmich Index); orbital semicircles are interrupted by one supraocular scales in both sides, but the rest is formed by ten scales on each side; 6–7 supraoculars (left-right); six superciliary scales. Frontal area is divided into three scales (one posterior, one middle and one anterior). Two scales between the nasal and the canthal. Preocular separated from the lorilabials by a single loreal scale. Nasal separated from rostral by one scale, surrounded by seven scales. One row of lorilabials between the supralabials and the subocular; seven supralabials, the fifth is curved upward without contacting the subocular; six infralabial scales. Mental scale is pentagonal, in contact with four scales; four pairs of postmental shields, the second is separated by two scales. Temporal scales are subimbricated and smooth or slightly keeled. Eleven temporal scales between the level of superciliary scales and the commissure of the mouth. Two projecting scales on the anterior edge of the ear, which do not cover the auditory meatus. Auricular scale is wide and restricted to the upper third of the meatus; 44 gulars between the auditory meatuses. Antehumeral fold and “Y” shaped lateral neck fold. Developed dorsolateral fold. Midbody scales: 94. Dorsal scales are rhomboidal, slightly keeled, without mucrons, subimbricate and with interstitial granules. Dorsal scales are similar in size than ventral ones. Dorsal scales: 89. Ventral scales are rhomboidal, smooth, imbricate, and without interstitial granules. Ventral scales: 124. Three precloacal pores. Hemipenial bulges are evident. The suprafemoral scales are lanceolate, imbricate, and slightly keeled. Infrafemoral scales are lanceolate to rounded, smooth, and imbricate. Scales of the dorsal surface of the forearm are lanceolate to rounded, imbricate, and slightly keeled or smooth. Scales of the ventral surface of the forearm are rounded, smooth, and subimbricate. The dorsal scales of the first third of the tail are rhomboidal to lanceolate, subimbricate or juxtaposed, keeled and with interstitial granules. The ventral scales of the tail vary from rhomboidal to triangular, and are imbricate and smooth. Lamellae of the fingers: I: 10, II: 14, III: 22, IV: 24 and V: 15. Lamellae of the toes: I: 11, II: 16, III: 22, IV: 32 and V: 19.

#### Coloration in life.

Light brown head, with dark brown spots in the parietal area and in the posterior nasal area. The snout is olive. Temporal area is light brown. Subocular area and cheeks are slightly lighter than temporal area. The subocular is immaculate. Background color of the dorsum, limbs, and tail is light brown. The vertebral zone of the dorsum is slightly darker than rest, but without forming an occipital stripe. The only dorsal design is a series of white dots, formed by 1–3 white scales, running from the posterior half of the trunk to the first third of the tail. The tail is immaculate. Ventrally, the throat, belly, limbs and the tail are whitish pearly. Thighs and cloaca have a little yellowish coloration. Precloacal pores are orange.

#### Variation.

Despite four field campaigns, no additional males were found. Variation in measures refer to the six female paratypes: SVL: 66.2–69.6 mm. Axilla-groin distance: 27.4–30.2 mm. Head length: 13.5–15.1 mm. Head width: 10.7–11.4 mm. Head height: 6.4–7.6 mm. Foot length: 18.0–21.5 mm. Leg length: 36.5–44.7 mm. Hand length: 9.4–11.7 mm. Arm length: 21.1–26.7 mm. Tail length: 84–110 (n = 3; autotomized in the rest). Relation tail length/SVL = 1.2–1.7. Although more data on males are required, there is no sexual size dimorphism in the *Liolaemus elongatus* clade species (Avila et al. 2012).

Scale number variation in *Liolaemus janequeoae* (all specimens) is as follows. Midbody scales: 82–98 (91.6 ±5.5). Dorsal scales: 77–89 (85.0 ±4.2). Ventral scales 124–132 (128.6 ±3.5). Fourth finger lamellae: 22-24 (23.5 ±0.8). Fourth toe lamellae: 28–32 (29.5 ±1.4). Supralabial scales: 6–8 (7.4 ±0.8). Infralabial scales: 5–6 (5.3 ±0.5). Interparietal scale is pentagonal or hexagonal, bordered by 5–9 scales (6.6 ±1.7). The interparietal is smaller than the parietals. The nasal is in contact with the rostral in 28.6% of specimens.

Females have a very similar color pattern to the male holotype but without dorsal white dots or yellowish coloration on the thighs and cloaca. One female has four series of black dots (formed by 1–3 black scales) on the dorsum: two on the paravertebral fields (running from the head to the first third of the tail) and two on the dorsolateral area (running from the head to the middle of the trunk).

#### Etymology.

This species is named after Janequeo, a prominent Lonko (tribal chief) of Mapuche-Pehuenche origins. She fought against colonial Spaniards in the Arauco war, carried out mainly in the Araucanía Region where *Liolaemus janequeoae* was discovered. It is believed that she became involved in the war after her partner (Lonko Hueputan) was captured and tortured to death. She played a leading role in the Battle of Fort Puchunqui, then retreating to Villarrica, where she disappeared.

#### Distribution and natural history.

**Figure 5.**
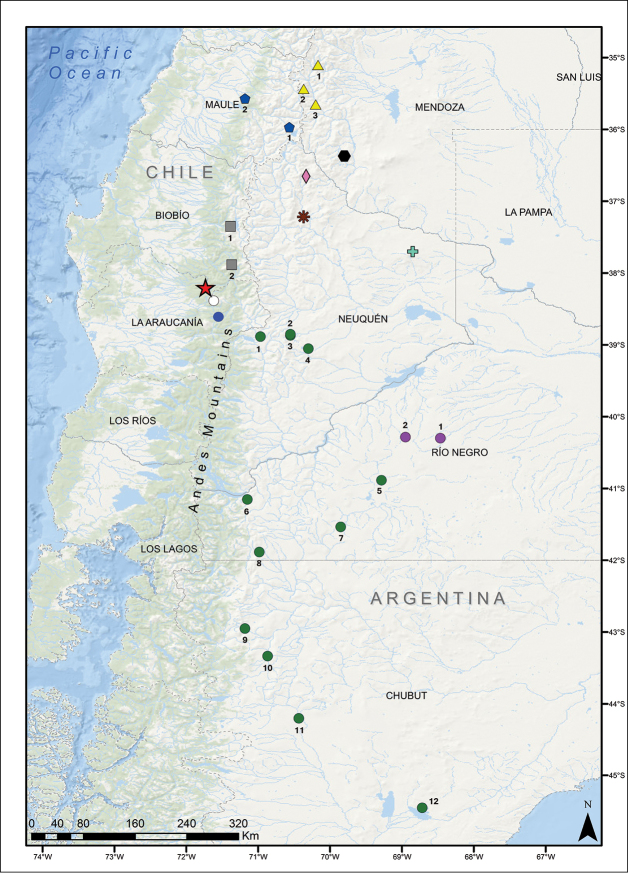
Distribution map for *Liolaemus janequeoae* sp. n. with geographically proximate species of the *Liolaemus elongatus* clade. In the case of *Liolaemus elongatus* a sample for each locality was included in the phylogeny. Red star: *Liolaemus janequeoae* sp. n., Laguna Verde. Yellow triangles: *Liolaemus smaug* (1= near Las Leñas, 2= between Las Loicas and Peteroa Volcano, 3= near Las Loicas). Blue pentagon: *Liolaemus carlosgarini* (1= Maule Lagoon, 2= Lircay). Black hexagon: *Liolaemus choique* (Paso el Choique). Pink diamond: *Liolaemus antumalguen* (Domuyo Volcano). Brown asterisk: *Liolaemus burmeisteri* (Caepe Malal). Green cross: *Liolaemus crandalli* (Auca Mahuida Volcano). Gray squares: *Liolaemus scorialis* (1= Laja Lagoon, 2= La Mula Lagoon). White circle: *Liolaemus lonquimayensis* (Lonquimay Volcano). Pink circles: *Liolaemus shitan* (1= Estancia Piedras Blancas, type locality and 2= near Antonio del Cuy). Blue circle: *Liolaemus* cf. *elongatus* (Llaima Volcano). Green circles: *Liolaemus elongatus* (1= Pampa de Lonco Luan, 2= Primeros Pinos, 3= Portal La Atravesada, 4= Laguna Blanca, 5= near Ingeniero Jacobacci, 6= San Carlos de Bariloche, 7= Ojo de Agua, 8= El Maiten, 9= Esquel, 10= Tecka, 11= Gobernador Costa and 12= Los Manantiales).

Only known from the type locality at Laguna Verde (38°12'S - 71°44'W), approximately 13.5 km NW of the summit of the Tolhuaca volcano, Araucanía Region, Chile (Fig. 5).

At Laguna Verde, *Liolaemus janequeoae* was found between 1336–1397 masl. It inhabits the deciduous highland Andean forest (Gajardo 1994), consisting of *Araucaria araucana* and *Nothofagus dombeyi* (1397 masl). The shrubs are represented by *Chusquea culeou*, *Desfontainia spinosa*, *Drimys andina* and *Pseudopanax laetevirens*. At lower altitudes (1336 masl), the vegetation was dominated by *Araucaria araucana* and *Nothofagus pumilio*, with the presence of *Azara alpine*, *Chusquea culeou*, *Colletia hystrix*, *Lomatia hirsuta*, *Maytenus disticha*, *Myrceugenia chrysocarpa* and *Pernettya myrtilloides*. At lower altitudes where there are no *Araucaria araucana*, *Liolaemus janequeoae* was not found. It is a diurnal lizard of apparently low abundance. It was seen on rocks and climbing in trees.

*Liolaemus janequeoae* was found in syntopy with *Liolaemus septentrionalis* Pincheira-Donoso and Núñez, 2005; *Liolaemus tenuis* (Duméril & Bibron, 1837); *Pristidactylus torquatus* (Philippi, 1861) and the second new species described below. In this zone, it was also recorded the presence of *Tachymenis chilensis* (Schlegel, 1837).

The intestinal content of one specimen (paratype) was examined and remnants of insects and several nematodes were found. At the date of capture (January 5) two females had two and three embryos each. All other females have only several small oocytes.

### Liolaemus leftrarui sp. n.

Taxon classificationAnimaliaSquamataLiolaemidae

http://zoobank.org/71CE0862-31F7-4ADD-B977-F00479198873

Proposed standard English name: Leftraru`s Lizard

Proposed standard Spanish name: Lagarto de Leftraru

Figure 6

#### Holotype.

**Figure 6.**
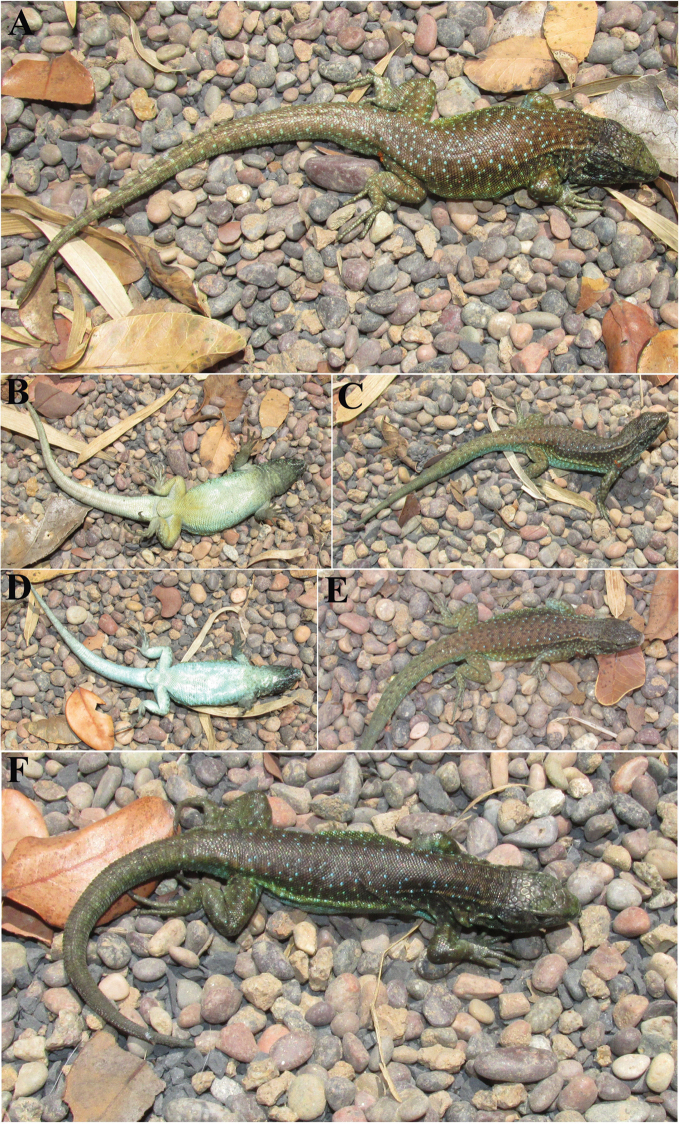
*Liolaemus leftrarui* sp. n. **A** and **B** Holotype, male **C** Dorsal and **D** ventral view of Paratype, female **E** and **F** Paratypes, females.

SSUC Re 646 (Fig. 6a, b). Male collected at Laguna Verde (38°12'S - 71°44'W, 1405 masl), approximately 13.5 km NW of the summit of the Tolhuaca volcano, Araucanía Region, Chile. Collected by J. Troncoso-Palacios, F. Urra and H. Díaz. January 5, 2014.

#### Paratypes.

SSUC Re 647–48, 716 (Fig. 6). Three females. Same data as the holotype. SSUC Re 732–734. Two males and one female. Near Lagunillas, Araucanía Region, Chile (38°12'S - 71°46'W, 1483 masl), approximately 4 km NW from the type locality. Collected by J. Troncoso-Palacios & E. Villarroel. September, 2016.

#### Diagnosis.

*Liolaemus leftrarui* is closely related to *Liolaemus villaricensis*. This species is characterized by 1) lack of precloacal pores in either sex, 2) large size *Liolaemus* (max. SVL = 81.8 mm), 3) high amount of midbody scales (80–88), 4) light blue dots on the dorsum, and 5) absence of ventral melanism. We provide a diagnosis in regards to *Liolaemus villaricensis*, plus four unrelated species that occur geographically near to *Liolaemus leftrarui* and that also feature the absence of precloacal pores. Based on seven specimens.

*Liolaemus leftrarui* has more dorsal scales than *Liolaemus villaricensis* (77–87 vs. 80–89) (t = -2.5, DF = 11, P < 0.05). Moreover, *Liolaemus villaricensis* has a marked lateral black band and a fragmented vertebral stripe, whereas in *Liolaemus leftrarui* these two color features are inconspicuous or less marked than in *Liolaemus villaricensis*. *Liolaemus villaricensis* has no light blue dots, which are in all specimens of *Liolaemus leftrarui*. Finally, although they are sister species, the average uncorrected pairwise distance between the two taxa is 7.3%, more than double that value proposed for identification of candidate species in *Liolaemus*. Additionally, PCA results show that both species only marginally overlap in morphological space when ellipses are generated with the two first PCs (Fig. 3).

*Liolaemus leftrarui* is larger (max. SVL = 81.8 mm) than *Liolaemus coeruleus* (males SVL = 58.7 ± 3.2 mm; females SVL = 58.2 ± 2.8 mm) and *Liolaemus neuquensis* (males SVL = 57.4 ± 3.5 mm; females SVL = 58.2 ± 1.9 mm). Moreover, *Liolaemus coeruleus* males feature black ventral color and some *Liolaemus neuquensis* males also feature a black ventral color, a feature absent in *Liolaemus leftrarui*. Females of *Liolaemus coeruleus* and *Liolaemus neuquensis* have a brown dorsal color, but females of *Liolaemus leftrarui* have a bluish brown dorsal color. Finally, in our phylogeny *Liolaemus neuquensis* is not closely related to *Liolaemus leftrarui* and although we have no molecular data for *Liolaemus coeruleus*, this last species and *Liolaemus neuquensis* are probably conspecific (Avila et al. 2003).

*Liolaemus leftrarui* has more midbody scales (80–88 vs. 67–81) than *Liolaemus punmahuida*. Dorsal color in *Liolaemus punmahuida* is ochre and this species is patternless, whereas *Liolaemus leftrarui* has brown dorsal color with dispersed light blue dots. *Liolaemus punmahuida* has reddish color around the cloaca, feature absent in *Liolaemus leftrarui*. The species are not closely related according to our phylogeny.

*Liolaemus leftrarui* differs from *Liolaemus tregenzai* in that this last species features black color on the throat, chest and abdomen of males and gray color on the throat, chest and abdomen of females, features totally absent in *Liolaemus leftrarui*. The species are not closely related according to our phylogeny.

#### Description of holotype.

Adult male. SVL: 81.7 mm. Tail length: 102.9 mm (not autotomized). Axilla-groin length: 35.4 mm. Head length: 20.1 mm. Head width (distance between the two ear openings): 16.9 mm. Head height (at the level of ear openings): 10.8 mm. Forelimb length: 26.5 mm. Hindlimb length: 46.0 mm. Foot length: 21.8 mm. Hand length: 13.6 mm. Rostral scale wider (4.3 mm) than high (1.6 mm). Subocular length: 5.7 mm. Fourth supralabial length: 3.4 mm. Neck width: 16.2 mm. Interorbital distance: 7.2 mm. Internasal distance: 3.0 mm. Body width: 27.2 mm. Meatus width: 1.0 mm. Meatus height: 3.3 mm.

Two postrostrals. Four internasals. Pentagonal interparietal scale, with a central, small, and whitish ‘‘parietal eye’’ in the center. Interparietal scale is similar in size to parietal one, surrounded by other six scales; seven scales between interparietal scale and rostral; twelve scales between occiput and rostral; orbital semicircle is incomplete in the right side and complete in the left side (formed by 12 scales); 5–4 supraoculars (left-right); five superciliary scales. Frontal area is divided into three scales (two posterior and one anterior). Remarkably, only one scale between the nasal and the canthal. Preocular separated from the lorilabials by a single loreal scale. Nasal in contact with the rostral, surrounded by seven scales. One row of lorilabials between the supralabials and the subocular; six supralabials, the fourth is curved upward without contacting the subocular; five infralabial scales. Mental scale is pentagonal, in contact with four scales; five pairs of postmental shields, the second is separated by two scales. Temporal scales are subimbricate and smooth, very few are slightly keeled. Eight temporal scales between the level of superciliary scales and the level of the commissure of the mouth. Two enlarged projecting scales on the anterior edge of the ear, which do not cover the auditory meatus. Auricular scale is wide and restricted to the upper third of the meatus; 42 gulars between the auditory meatuses. Antehumeral fold and “Y” shaped lateral neck fold. Present inconspicuous ventrolateral fold. Midbody scales 86. Dorsal scales are rounded to lanceolate, slightly keeled, without mucrons, imbricate and with some interstitial granules. Dorsal scales are smaller than ventral ones. Dorsal scales 81. Ventral scales are rhomboidal to rounded, smooth, imbricate, and without interstitial granules. Ventral scales 118. There are no precloacal pores. Hemipenial bulges are evident. The suprafemoral scales are lanceolate, imbricate, and smooth or slightly keeled. Infrafemoral scales are rounded, smooth, and imbricate. Scales of the dorsal surface of the forearm are rounded, imbricate, and slightly keeled or smooth. Scales of the ventral surface of the forearm are rounded, smooth, juxtaposed or subimbricate with interstitial granules. The dorsal scales of the tail are rhomboidal, imbricate, keeled and some with mucrons. The ventral scales of the tail vary from rhomboidal to triangular, and are imbricate and smooth. Lamellae of the fingers: I: 12, II: 14, III: 20, IV: 22 and V: 15. Lamellae of the toes: I: 11, II: 16, III: 21, IV: 27 and V: 18.

#### Coloration in life.

Brown head, with dispersed dark brown spots. Occipital area of the head is dark brown; temporal area is brown with three dark brown stripes and some dispersed light blue scales. Ocular area, snout and cheeks are light green. Subocular scale is light blue with two dark brown vertical lines, one in the middle and other in the anterior edge. Background color of the dorsum is brown. Inconspicuous dorsolateral light brown stripe (two scales of wide) running from the occiput level to the level of the axilla. Dark brown spots dispersed on the dorsum, without forming an occipital band, but forming three lines on the neck; one of which (middle) forms an inconspicuous vertebral stripe on the dorsum. Several light blue dots dispersed on the dorsum (each corresponds to one scale). Inconspicuous dark brown lateral band with dispersed light blue scales. Below lateral band, flanks are yellowish. Limbs are brown with light green and few dispersed light blue scales. Tail is brown with dispersed light green scales and dark brown vertebral line. Ventrally, the throat is dark green, darker towards the tip of the snout. Belly and the tail are light green. Rear portion of belly, cloaca, chest and thighs have a yellowish coloration. Palms are dark brown and soles are light brown.

#### Variation.

Variation in three males (including the holotype): SVL: 76.1–81.8 mm. Axilla-groin distance: 33.2–35.7 mm. Head length: 17.9–20.1 mm. Head width: 14.6–16.9 mm. Head height: 9.3–10.8 mm. Foot length: 20.2–21.8 mm. Leg length: 42.7–46.0 mm. Hand length: 12.0–13.6 mm. Arm length: 26.0–27.3 mm. Tail autotomized in all male paratypes. Variation in four female paratypes is as follows: SVL: 60.5–68.2 mm. Axilla-groin distance: 26.4–30.1 mm. Head length: 13.2–15.0 mm. Head width: 9.7–12.0 mm. Head height: 6.3–7.0 mm. Foot length: 17.4–17.9 mm. Leg length: 32.5–38.2 mm. Hand length: 10.1–11.1 mm. Arm length: 20.5–21.2 mm. Tail autotomized in all females.

Scale number variation in *Liolaemus leftrarui* (all specimens) is as follows. Midbody scales: 80–88 (84.3 ±3.5). Dorsal scales: 77–87 (81.3 ±3.6). Ventral scales 108–123 (115.3 ±5.8). Fourth finger lamellae: 20-23 (21.9 ±1.1). Fourth toe lamellae: 27–30 (28.1 ±1.3). Supralabial scales: 6–7 (6.4 ±0.5). Infralabial scales: 4–5 (4.7 ±0.5). Holotype has only one scale between the nasal and the canthal, but paratypes have two, as usual in the genus *Liolaemus*. No precloacal pores in the males and no vestigial precloacal pores in the females, which is rare in *Liolaemus*. Interparietal scale is quadrangular, pentagonal, hexagonal or heptagonal, bordered by 5–7 scales (5.7 ±0.8). The interparietal is similar size or smaller than the parietals. The canthal is in contact with the rostral in all specimens.

Paratype males have similar coloration pattern to the holotype with variation only in shade. Females have similar coloration pattern to the holotype, but with some differences such as: the dark brown color on the occipital area is less marked or absent; the dark brown lateral band (inconspicuous in the holotype) is marked in some females; the dark brown vertebral stripe of the tail is inconspicuous or absent in females; the ventral color is light green or light blue; the throat is reticulated in one female; the yellowish color on the rear portion of belly and the cloaca is less marked or absent in females.

#### Etymology.

This species is named after Leftraru, the most prominent Lonko (tribal chief) of the Mapuche people, who fought against colonial Spaniards in the Arauco war, carried out mainly in the Araucanía Region where we discovered *Liolaemus leftrarui*. He was captured when he was eleven by Pedro de Valdivia (Governor of the Kingdom of Chile) and became his personal servant. He learned the military strategy of the Spanish and then escaped. Later, he ambushed and killed Valdivia, and won the most remarkable victories over the Spaniards. Finally, he was surrounded and died in battle.

#### Distribution and natural history.

**Figure 7.**
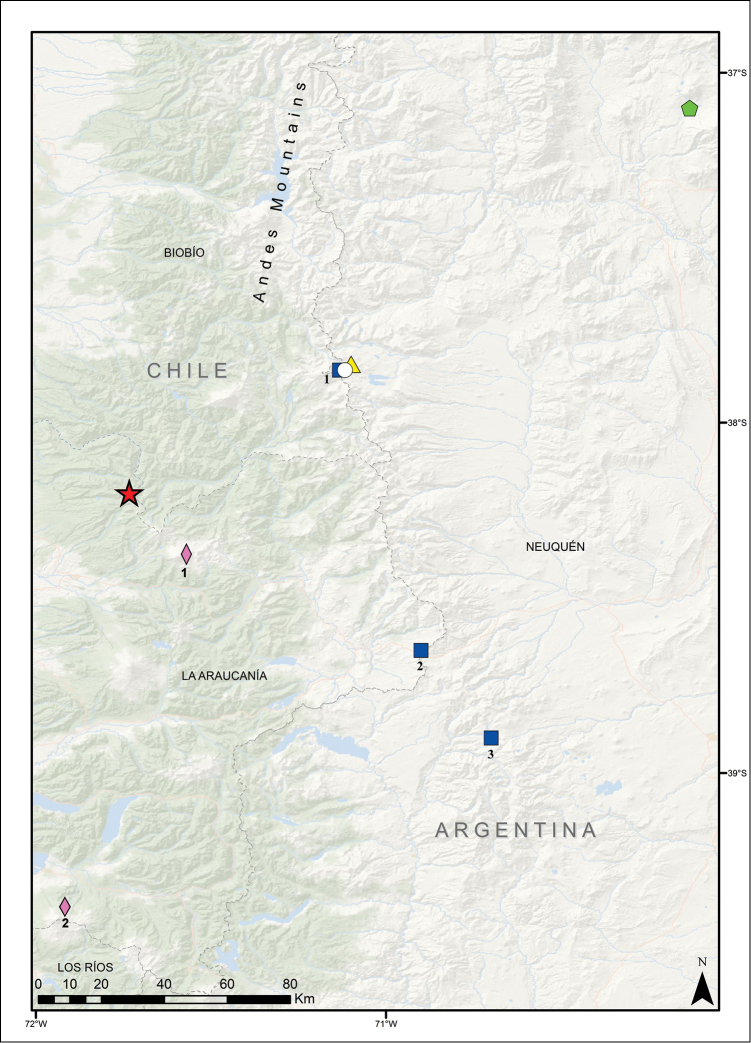
Distribution map for *Liolaemus leftrarui* sp. n. with closely related *Liolaemus villaricensis* and geographically proximate species that feature a lack of precloacal pores. Red star: *Liolaemus leftrarui* sp. n. (Laguna Verde and Lagunillas). Pink diamond: *Liolaemus villaricensis* (1= Lonquimay Volcano, 2 = Villarrica Volcano). Blue squares: *Liolaemus coeruleus* (1= Copahue, 2= Pino Hachado, 3= Primeros Pinos). White circle: *Liolaemus tregenzai* (Copahue). Yellow triangle: *Liolaemus neuquensis* (Copahue). Green pentagon: *Liolaemus punmahuida* (Tromen Volcano).

Known from two localities: 1) the type locality at Laguna Verde (38°12'S - 71°44'W), approximately 13.5 km NW of the summit of the Tolhuaca volcano, Araucanía Region, Chile (Fig. 7). At Laguna Verde, *Liolaemus leftrarui* was found between 1336–1397 masl. Vegetation is the same described for the habitat of *Liolaemus janequeoae*. At lower altitudes where there are no *Araucaria araucana*, *Liolaemus leftrarui* was not found. 2) Near Lagunillas (38°12'S - 71°46'W, 1483 masl), approximately 4 km NW from Laguna Verde, in the *Araucaria araucana* forest. It is probable that the distribution of *Liolaemus leftrarui* could extend to Lagunillas (1700 masl) but in September (date of collection) this area is covered with snow and no specimens were found. Remarkably, *Liolaemus janequeoae* was not found near Lagunillas. *Liolaemus leftrarui* is a diurnal lizard of apparently low abundance at both localities. It was seen on rocks and trees (in Laguna Verde), clambering to approximately 5 m aboveground in trees when threatened. Near Lagunillas it was seen only in fallen trees.

*Liolaemus leftrarui* was found in syntopy with *Liolaemus septentrionalis*, *Liolaemus tenuis*, *Liolaemus janequeoae* and *Pristidactylus torquatus* at the type locality. Near Lagunillas it was found in syntopy with *Liolaemus septentrionalis* and *Liolaemus tenuis*. In this zone the presence of *Tachymenis chilensis* was also recorded.

The intestinal contents of one specimen from the type locality was examined and revealed the remnants of insects. No plant remains were found. One specimen from near Lagunillas had several nematodes in the intestines. The females collected in January had several small oocytes but the female collected in September carried one embryo.

## Discussion

The diversity of the Chilean members of the *Liolaemus elongatus-kriegi* complex has been largely underestimated. Recent expeditions to seldom explored highlands and the revision of the taxonomic status of some populations has led to the description of several new species (Escobar-Huerta et al. 2015, Esquerré et al. 2013, 2014, Núñez 2007, Troncoso-Palacios et al. 2015). In fact, it has been thought that Chilean species of the *Liolaemus elongatus-kriegi* complex have a small distribution in central Chile (Morando et al. 2003), but currently it is known that this group of lizards is widely distributed in central and southern Chile, and it is also probable that some populations under study could be described as new species in the future (Troncoso-Palacios unpublished data).

The new species, *Liolaemus janequeoae*, was found to be member of the *Liolaemus elongatus* clade and the sister species of the clade formed of *Liolaemus elongatus* + *Liolaemus lonquimayensis* + *Liolaemus shitan*, but these findings are preliminary, since there are no *Cyt-b* sequences in GenBank for some species currently assigned to the *Liolaemus elongatus* clade (*Liolaemus carlosgarini*, *Liolaemus crandalli* and *Liolaemus scorialis*). A future study with additional species could yield a different topology. Moreover, a limitation in our study is the use of a single mtDNA marker, one limitation also shared by almost all recent descriptions of *Liolaemus* (*sensu stricto*). For example, hybridization and introgression have been found in closely related species of *Liolaemus* (Olave et al. 2011) and a future study of the species described here using nuclear markers would be greatly desirable. Besides, the clade formed by *Liolaemus elongatus* + *Liolaemus lonquimayensis* + *Liolaemus shitan* requires a deeper analysis. A sample of *Liolaemus shitan* does not form a monophyletic haploclade with respect to *Liolaemus elongates*. A work published previously to the description of this species showed that this “dark phenotype” from San Antonio del Cuy (25 de Mayo, Argentina) is not genetically distinctive enough to consider it as candidate species (Morando et al. 2003: 178) and it has been suggested as possible synonym of *Liolaemus elongatus* by Avila et al. (2015). However, since there are currently no DNA data for *Liolaemus shitan* from the type locality (Estancia Piedras Blancas, 25 de Mayo), we tentatively accept this species as valid. In regard to *Liolaemus lonquimayensis*, we believe that the relationship between this taxon and *Liolaemus elongatus* is not solved. The distinction between them is based in two features: absence of precloacal pores in the males of *Liolaemus lonquimayensis* and the fact that the four type specimens of *Liolaemus lonquimayensis* form a clade separated from *Liolaemus elongatus* samples (Escobar-Huerta et al. 2015). However, Escobar-Huerta et al. (2015) used three sequences of *Liolaemus elongatus* in their phylogeny (BYU 47101, MVZ 232399 and BYU 47092). We used thirteen sequences of *Liolaemus elongatus* and one sequence of *Liolaemus shitan* and obtained a different result, *Liolaemus lonquimayensis* does not form a distinctive clade from *Liolaemus elongatus* (Figs 1 and 2). In this work, we include in the PCA analysis specimens of *Liolaemus* cf. *elongatus* from Llaima Volcano, located between the type locality of *Liolaemus lonquimayensis* and the northern limit of *Liolaemus elongatus* (Morando et al. 2003). Based on coloration and morphology, these specimens can be assigned to *Liolaemus elongatus*, although there are no molecular data to confirm this.

The second new species that is described here, *Liolaemus leftrarui*, is notable for the absence of precloacal pores and its light blue dorsal dots, because precloacal pores in males a typical feature in *Liolaemus* (Esquerré et al. 2013). The absence of precloacal pores also occurs in *Liolaemus villaricensis* (Torres-Pérez et al. 2009), the most closely related species to *Liolaemus leftrarui*. However, molecular evidence indicates that the species of the subgenus *Liolaemus* with complete absence of precloacal pores in males are paraphyletic and not monophyletic, as has been previously proposed (Cei and Videla 2003). For example, in our phylogeny *Liolaemus leftrarui*, *Liolaemus neuquensis*, *Liolaemus punmahuida* and *Liolaemus tregenzai* do not constitute a monophyletic group and none of them is the sister species of the others (although precloacal pores are lacking in all these species). Pincheira-Donoso and Núñez (2005) recorded *Liolaemus villaricensis* from Lonquimay volcano, but no specimens were deposited in an institutional collection. Here we add a second record of this species from this locality (SSUC Re 729–31).

Certainly, there is still much to be discovered about the diversity of the species of *Liolaemus* in southern and central Chile, especially in the *Liolaemus elongatus-kriegi* complex and the species related to *Liolaemus villaricensis*, for which several taxonomic issues still remain unsolved.

## Supplementary Material

XML Treatment for Liolaemus janequeoae

XML Treatment for Liolaemus leftrarui

## Supplementary materials

Supplementary material 1 Appendices Data type: Microsoft Word (docx) Explanation note:
**Appendix I**: Museum codes of the specimens examined.
**Appendix II**: GenBank accession numbers of the specimens used for phylogenetic analysis.
**Appendix III**: Eigenvalues, the percentage of the total variance and the cumulative percentage of variance in each of the 13 PCs found by the PCA.
**Appendix IV**: Correlation of each variable with each of the first three PCs. This dataset is made available under the Open Database License (http://opendatacommons.org/licenses/odbl/1.0/). The Open Database License (ODbL) is a license agreement intended to allow users to freely share, modify, and use this Dataset while maintaining this same freedom for others, provided that the original source and author(s) are credited.Jaime Troncoso-Palacios, Hugo A. Diaz, German I. Puas, Edvin Riveros-Riffo, Alvaro A. Elorza
