# Supplementary material for: Two new Liolaemus lizards from the Andean highlands of Southern Chile (Squamata, Iguania, Liolaemidae)
Source: Zookeys. 2016 Nov 16;(632):121–46. doi: 10.3897/zookeys.632.9528 (PMC5126550; doi:10.3897/zookeys.632.9528)
Supplement: Supplementary material 1 — Appendices [file zookeys-632-121-s001.docx]

**Appendix I**

Specimens examined. Museum codes are as follow: MZUC (Colección del Museo de Zoología de la Universidad de Concepción), MRC (Museo de Historia Natural de Concepción) and SSUC (Colección Patricio Sánchez Reyes, Pontificia Universidad Católica de Chile).

*Liolaemus carlosgarini*

SSUC Re 181–189, 349. Road to Maule Lagoon, Maule Region. F. Ferri coll. February 20, 2011.

*Liolaemus cf. elongatus*

SSUC Re 545–46, 618–21. Eight km east of the summit of the Llaima volcano, Araucanía Region, Chile. Collected by J. Troncoso-Palacios & D. Esquerré. December 9, 2012.

*Liolaemus janequeoae*

SSUC Re 712–14. Laguna Verde, approximately 13.5 km NW of the summit of the Tolhuaca volcano, Araucanía Region, Chile. J. Troncoso-Palacios & Edvin Riveros-Riffo colls. January 15, 2016. SSUC Re 715. Laguna Verde, approximately 13.5 km NW of the summit of the Tolhuaca volcano, Araucanía Region, Chile. Edvin Riveros-Riffo coll. February 18, 2015. SSUC Re 649–51. Laguna Verde, approximately 13.5 km NW of the summit of the Tolhuaca volcano, Araucanía Region, Chile. J. Troncoso-Palacios, F. Urra & H. Díaz colls. January 5, 2014.

*Liolaemus leftrarui*

SSUC Re 646–48, 716. Laguna Verde, approximately 13.5 km NW of the summit of the Tolhuaca volcano, Araucanía Region, Chile. J. Troncoso-Palacios, F. Urra & H. Díaz colls. January 5, 2014. SSUC Re 732–734. Near Lagunillas, Araucanía Region, Chile. J. Troncoso-Palacios & E. Villarroel colls. September, 2016.

*Liolaemus scorialis*

SSUC Re SSUC Re 612–17, 680. 7 km NW of the summit of the Antuco volcano, near the Laja Lagoon, Biobío Region, Chile. J. Troncoso-Palacios, F. Urra & H. Díaz colls. January 8, 2014. MRC 675, 677, 680, 682. La Mula Lagoon, Ralco National Reserve. Unknown coll. December 1, 2001.

*Liolaemus villaricensis*

MZUC 30590, 30592–93. Villarrica volcano. F. Torres-Pérez coll. January 2, 1986. SSUC Re 729–31. Lonquimay volcano. J. Troncoso-Palacios & E. Riveros colls. January 13, 2016.

**Appendix II**

Specimens used for phylogenetic analysis.

DNA sequences obtained in this study. *Liolaemus janequeoi.* SSUC Re 650 (KU936835) and SSUC Re 651 (KU936836). *L. leftrarui.* SSUC Re 648 (KU936837).

DNA sequences obtained from GenBank. 1. *Liolaemus elongatus* clade. *L*. *antumalguen.* KP121325 and KP121335. *L. burmeisteri.* KP121327, KP121328. *L. choique.* KP121329. *L. elongatus.* AY173798, AY173801, AY173803, AY173806, AY173809, AY173815, AY173818, AY173826, AY173827, AY173848, AY173852, AY173853, AY173855. *L. shitan*. AY173840. *L. smaug*. AY173830, AY173832. *L. sp*. Chillán. AY529901, AY730673, AY730668, AY850621, AY529902, AY730669. *L. sp*. 6. AY367799. *L. sp*. 7. AY367799, AY367799. 2. *Liolaemus kriegi* clade. *L. buergeri*. KJ494070, KJ494079, KJ494080. *L. kriegi.* AY173802, AY173814, KJ494012, KJ494150, KJ494155, KJ494188, KJ494190, KJ494191. *L. tregenzai*. AY367817, KJ494036, KJ494037, KJ494038, KJ494039, KJ494040, KJ494230. *L. zabalai*. KJ494056, KJ494057, KJ494059, KJ494074, KJ494085, KJ494086. 3. *Liolaemus petrophilus* clade. *L. austromendocinus.* AY173838. *L. capillitas.* AY173844. *L. dicktracy.* AY367816. *L. gununakuna.* AY367807, AY173859. *L. parvus*. AY173836. *L. petrophilus*. JN847103, JN847211, AY173796. *L. talampaya.* AY173797. *L. tulkas*. AY367813. *L. umbrifer*. AY367814. 2. *Liolaemus alticolor-bibronii* group. *L. abdalai*. JN410525. *L. alticolor*. KF923659, KF923660. *L. incaicus*. KF923657, KF923658. *L. ramirezae*. DQ989772. *L. robertmertensi*. DQ989769. *L. saxatilis*. JN410527, JN410553. Other out groups. *L. bellii*. KU095830, KU095831. *L. cf. chillanensis*. AY529903, AY529904, AY730670, AY850623, AY850624, AY850625. *L. chiliensis*. DQ989785, EU649245. *L. cyanogaster*. DQ989786. *L. monticola.* AY850619, AY850616, AY851724, AY851718, AY851726, AY851713, AY851708, AY851710. *L. neuquensis.* AY173828. *L. pictus*. AY173795. *L. punmahuida*. AY173824. *L. villaricesis*. AY850626, AY850627, AY850628, AY850629, AY730671, AY730672, AY529905, AY529906, DQ989787. *Phymaturus vociferator*. JX969016. *P. felixi.* JX969044.

**Appendix III**

Eigenvalues, the percentage of the total variance and the cumulative percentage of variance in each of the 13 PCs found by the PCA.

|  | **PC1** | **PC2** | **PC3** | **PC4** | **PC5** | **PC6** | **PC7** | **PC8** | **PC9** | **PC10** | **PC11** | **PC12** | **PC13** |
| --- | --- | --- | --- | --- | --- | --- | --- | --- | --- | --- | --- | --- | --- |
| Eigenvalue | 6.40 | 1.77 | 1.48 | 0.87 | 0.75 | 0.59 | 0.49 | 0.21 | 0.15 | 0.11 | 0.08 | 0.03 | 0.01 |
| % of var. | 49.29 | 13.61 | 11.42 | 6.69 | 5.79 | 4.55 | 3.80 | 1.66 | 1.22 | 0.86 | 0.62 | 0.29 | 0.14 |
| Cum. % of var. | 49.29 | 62.91 | 74.33 | 81.03 | 86.82 | 91.37 | 95.18 | 96.84 | 98.07 | 98.93 | 99.56 | 99.85 | 100 |

**Appendix IV**

Correlation of each variable with each of the first three PCs.

|  | **PC1** | **PC2** | **PC3** |
| --- | --- | --- | --- |
| Snout-vent length (SVL) | 0.977 | 0.007 | 0.043 |
| Head length (HL) | 0.959 | -0.221 | 0.038 |
| Head height (HH) | 0.866 | -0.298 | 0.029 |
| Head width (HW) | 0.917 | -0.258 | 0.022 |
| Axilla-groin distance (AGD) | 0.926 | -0.030 | 0.096 |
| Foot length (FL) | 0.916 | 0.224 | 0.037 |
| Arm length (AL) | 0.880 | -0.015 | 0.300 |
| Scales around mid-body (SAMB) | -0.390 | -0.332 | 0.645 |
| Ventral scales (VS) | -0.351 | 0.052 | 0.682 |
| Dorsal scales (DS) | -0.279 | -0.517 | 0.538 |
| Supralabials | 0.085 | 0.708 | 0.302 |
| Infralabials | 0.285 | 0.420 | 0.234 |
| 4^th^ Toe lamellae | 0.146 | 0.667 | 0.246 |
